# Mathematical Modeling Predicts Enhanced Growth of X-Ray Irradiated Pigmented Fungi

**DOI:** 10.1371/journal.pone.0085561

**Published:** 2014-01-15

**Authors:** Igor Shuryak, Ruth A. Bryan, Joshua D. Nosanchuk, Ekaterina Dadachova

**Affiliations:** 1 Center for Radiological Research, Columbia University, New York, New York, United States of America; 2 Department of Radiology, Albert Einstein College of Medicine, Bronx, New York, United States of America; 3 Department of Medicine, Albert Einstein College of Medicine, Bronx, New York, United States of America; 4 Department of Microbiology and Immunology, Albert Einstein College of Medicine, Bronx, New York, United States of America; University of Birmingham, United Kingdom

## Abstract

Ionizing radiation is known for its cytotoxic and mutagenic properties. However, recent evidence suggests that chronic sub-lethal irradiation stimulates the growth of melanin-pigmented (melanized) fungi, supporting the hypothesis that interactions between melanin and ionizing photons generate energy useful for fungal growth, and/or regulate growth-promoting genes. There are no quantitative models of how fungal proliferation is affected by ionizing photon energy, dose rate, and presence versus absence of melanin on the same genetic background. Here we present such a model, which we test using experimental data on melanin-modulated radiation-induced proliferation enhancement in the fungus *Cryptococcus neoformans*, exposed to two different peak energies (150 and 320 kVp) over a wide range of X-ray dose rates. Our analysis demonstrates that radiation-induced proliferation enhancement in *C. neoformans* behaves as a binary “on/off” phenomenon, which is triggered by dose rates <0.002 mGy/h, and stays in the “on” position. A competing dose rate-dependent growth inhibition becomes apparent at dose rates >5000 mGy/h. Proliferation enhancement of irradiated cells compared with unirradiated controls occurs at both X-ray peak energies, but its magnitude is modulated by X-ray peak energy and cell melanization. At dose rates <5000 mGy/h, both melanized and non-melanized cells exposed to 150 kVp X-rays, and non-melanized cells exposed to 320 kVp X-rays, all exhibit the same proliferation enhancement: on average, chronic irradiation stimulates each founder cell to produce 100 (95% CI: 83, 116) extra descendants over 48 hours. Interactions between melanin and 320 kVp X-rays result in a significant (2-tailed p-value = 4.8×10^−5^) additional increase in the number of radiation-induced descendants per founder cell: by 55 (95% CI: 29, 81). These results show that both melanin-dependent and melanin-independent mechanisms are involved in radiation-induced fungal growth enhancement, and implicate direct and/or indirect interactions of melanin with high energy ionizing photons as an important pro-proliferative factor.

## Introduction

Survival (and sometimes relative predominance) of melanized fungi in environments exposed to high levels of ionizing radiation has been reported for several decades, for example from a nuclear weapons test site in Nevada [1], and from a forest experimentally exposed to chronic irradiation at the Brookhaven National Laboratory [2]. Data from the zone contaminated by the Chernobyl nuclear power plant accident [3]–[7] revealed that certain melanized fungi survive chronic irradiation from multiple radionuclides (even within the destroyed nuclear power plant buildings), and that ionizing radiation can stimulate spore germination and attract hyphal growth in some of these fungal strains. Recent laboratory investigations confirm the conclusion that some melanized fungi are not only radioresistant, but exhibit enhanced proliferation during chronic sub-lethal irradiation [8]–[10]. These findings suggest that the biological effects of ionizing radiation are not limited to cell death, mutagenesis and carcinogenesis [11], but can include growth stimulation of certain life forms. Interactions of ionizing photons with fungal melanin represent an important candidate mechanism for pro-proliferative effects of radiation on melanized fungi [9], [12]–[15].

To quantitatively analyze the phenomenon of fungal growth modulation by chronic ionizing radiation exposure, we propose the following mathematical model: Q = A(e, t, m) – [B(e, t, m) x R]. Here Q is the predicted radiation effect on proliferation relative to background conditions (for example, the excess number of cells which descended from each founder cell due to radiation effects on proliferation), and R is radiation dose rate. The adjustable parameters (A and B) depend on X-ray peak energy (e), duration of irradiation (t), and cell melanization status (m). Parameter A represents radiation-induced growth enhancement, which is assumed to be a “binary” qualitative response triggered by very low dose rates, e.g. due to modulation of certain metabolic and cell cycle-related pathways by interactions between melanin and ionizing photons, by redox processes, and/or by responses to low levels of DNA damage. Parameter B represents radiation-induced growth inhibition, which is assumed to be caused by DNA damage response pathways and hence proportional to dose rate. This simple formalism provides a tractable tool for analyzing and mechanistically interpreting experimental data on the influence of chronic sub-lethal irradiation on fungal growth. In particular, the effects of cell melanization and other factors on the values of both model parameters (A and B) can be analyzed quantitatively and statistically.

## Results

**Figure 1:**
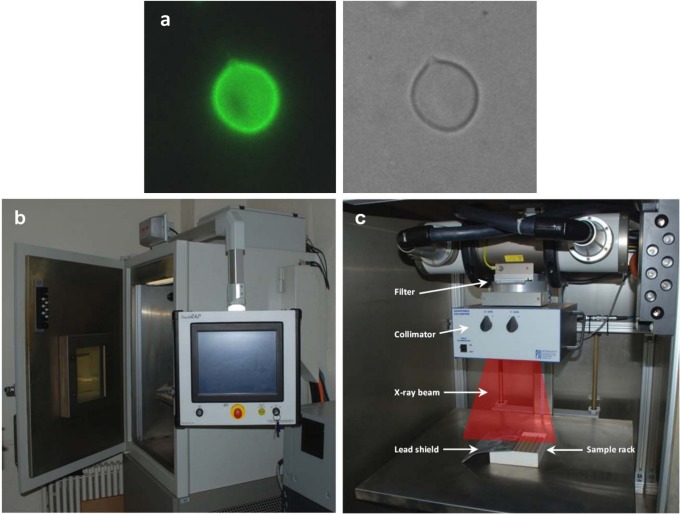
Fungal model system and experimental setup. (**a**) Melanized cell of the fungus *C. neoformans*: immunofluorescent image shows the melanin layer contiguous with the cell wall (left panel) and light microscopy image of the same cell (right panel). (**b–c**) External and internal views of the X-RAD 320 biological irradiator, which was used for continuous X-ray irradiation of *C. neoformans* cells. The cells were suspended in liquid medium in test tubes inserted into the sample rack. Radiation quality and dose rate were adjusted by varying the peak energy, current, filter, lead shielding thickness, and distance from the source.

To generate experimental data for testing model predictions, we selected the pathogenic fungus *Cryptococcus neoformans* (strain H99), which becomes melanized (Figure 1a) if a melanin precursor (L-DOPA in this case) is provided in the growth medium, and remains non-melanized otherwise, while the genetic background remains constant. Since melanin forms a contiguous layer with the cell wall (Figure 1a), incoming X-rays unavoidably pass through melanin before reaching the cytoplasm. We irradiated melanized and non-melanized *C. neoformans* cells under identical conditions with X-ray spectra of different peak energies (150 or 320 kVp) and a range of dose rates (0.002 to 5500 mGy/h) using the X-RAD 320 biological irradiator (Figure 1b–c).

### Analysis of Experimental Data

The effect of X-ray irradiation on the average number of descendants (Q_e_) produced by each founder cell during the exposure period was defined as follows: Q_e_ = (X_r_(i) – X_c_)/X_0_, where X_r_(i) is the number of colony-forming units per milliliter (CFU/ml) for each irradiated culture, X_c_ is the mean CFU/ml for corresponding unirradiated controls, and X_0_ is the mean CFU/ml at the start of irradiation. As described in the Materials and Methods section, Q_e_ is less sensitive to random inter-experimental variability than raw CFU/ml, because it represents the radiation-induced change in CFU/ml, normalized by the initial cell concentration. Therefore, Q_e_ values are more convenient than raw CFU/ml counts for detecting subtle effects of X-ray peak energy and melanization on cell proliferation, which are of main interest here.

**Figure 2:**
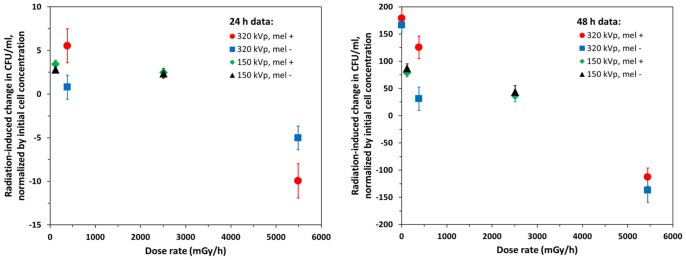
Effects of 24 and 48 hours of continuous X-ray irradiation on the proliferation of *C. neoformans*. The symbols represent mean values of the radiation-induced change (Q_e_) in CFU/ml (normalized by the initial cell concentration) for the same X-ray dose rate categories as in Table 1, plotted as function of mean dose rate for each category. Error bars represent standard errors. The abbreviations “mel +” and “mel –” represent melanized and non-melanized cells, respectively.

**Figure 3:**
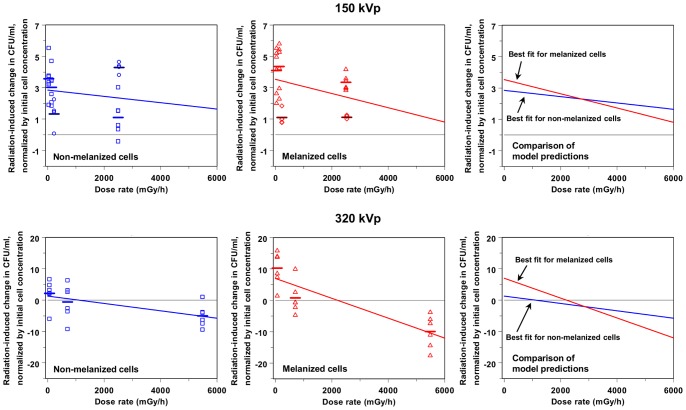
Analysis of the proliferation of *C. neoformans* continuously X-irradiated for 24 hours. The symbols represent radiation-induced change (Q_e_) in CFU/ml (normalized by the initial cell concentration) measured for melanized and non-melanized cells at each combination of X-ray peak energy (150 or 320 kVp) and dose rate. Different symbol colors, shapes, and bar colors represent different experiments. Horizontal bars indicate mean values from each experiment. The lines are best-fit predictions generated by the proposed model, Q = A(e, t, m) – [B(e, t, m) x R], which predicts the radiation effect on proliferation relative to background conditions (Q, shown on the y-axis), based on radiation dose rate (R shown on the x-axis), X-ray peak energy (e), duration of irradiation (t), and cell melanization status (m). Model parameter A represents radiation-induced growth enhancement at low dose rates, and parameter B represents radiation-induced growth inhibition, proportional to dose rate.

**Figure 4:**
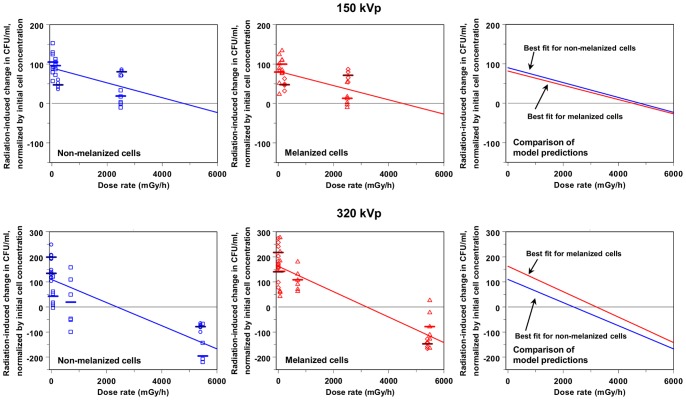
Analysis of the proliferation of *C. neoformans* continuously X-irradiated for 48 hours. Interpretations for the axes, symbols, and curves are the same as in Figure 3.

Chronic exposure to X-ray dose rates below 5000 mGy/h had a pro-proliferative effect on *C. neoformans*, resulting in extra descendants being produced by each founder cell. This tendency is shown by the positive mean Q_e_ values and their corresponding 95% confidence intervals for *C. neoformans* cultures exposed to dose rates in this range (Table 1 and Figure 2). Due to radiation-induced alteration of cell proliferation, each founder cell gave rise to an average of approximately 1–5 extra descendants over 24 hours of exposure, and to approximately 40–180 extra descendants over 48 hours (see Table 1 and data points in Figures 3–4).

**Table 1:** Effects of continuous X-ray irradiation with different peak energies and dose rates on the proliferation of *C. neoformans*.

Duration of irradiation (hours)	X-ray peak energy (kVp)	Cell melani-zation	Dose rate category (mGy/h)	Mean dose rate (mGy/h)	Radiation-induced change (Q_e_) in CFU/ml, normalized by the initial cell concentration	
					Mean	95% CIs
24	150	−	1–1000	121	2.81	2.16, 3.45
			2000–3000	2510	2.38	1.27, 3.49
		+	1–1000	121	3.45	2.57, 4.32
			2000–3000	2510	2.45	1.74, 3.17
	320	−	1–1000	383	0.78	−1.93, 3.49
			>5000	5490	−5.03	−7.67, −2.38
		+	1–1000	383	5.53	1.71, 9.34
			>5000	5490	−9.94	−13.79, −6.09
48	150	−	1–1000	121	87.2	71.4, 103.0
			2000–3000	2510	43.5	20.5, 66.4
		+	1–1000	121	79.2	63.6, 94.8
			2000–3000	2510	36.4	15.5, 57.3
	320	−	<1	0.12	166.4	141.6, 191.1
			1–1000	383	31.1	−10.1, 72.4
			>5000	5450	−137.0	−180.7, −93.2
		+	<1	0.12	178.9	147.1, 210.7
			1–1000	383	125.4	84.7, 166.1
			>5000	5450	−112.6	−145.3, −80.0

As discussed in the main text, radiation effects were quantified by calculating the radiation-induced change (Q_e_) in CFU/ml, normalized by the initial cell concentration, as follows: Q_e_ = (X_r_(i) – X_c_)/X_0_, where X_r_(i) is the CFU/ml for each irradiated culture, X_c_ is the mean CFU/ml for corresponding unirradiated controls, and X_0_ is the mean CFU/ml at the start of irradiation. Q_e_ represents the number of extra descendants produced by each founder cell due to the effects of irradiation on cell proliferation.

The modulation of radiation-induced growth enhancement by melanin was more subtle, and depended on X-ray peak energy. For 150 kVp X-ray exposures, there were no significant differences between the number of extra descendants per founder cell produced by non-melanized and by melanized *C. neoformans*. On average, each non-melanized founder cell produced 2.6 (range: −0.4, 5.5) extra descendants due to radiation effects over 24 hours, and 70 (range: −11, 153) extra descendants over 48 hours. Each melanized founder cell produced 3.1 (range: 0.8, 5.8) extra descendants over 24 hours, and 63 (range: −9, 150) extra descendants over 48 hours.

However, for 320 kVp X-ray exposures, differences between radiation responses of melanized and non-melanized cells began to emerge after 24 hours of irradiation, and reached statistical significance after 48 hours. On average, each non-melanized founder cell exposed to X-ray dose rates <5000 mGy/h produced 0.8 (range: −9.3, 6.6) extra descendants due to radiation effects over 24 hours, and each melanized founder cell produced 5.5 (range: −4.6, 16) extra descendants under the same conditions. Over a longer period of irradiation (48 hours), each non-melanized founder cell exposed to X-ray dose rates <5000 mGy/h produced 99 (range: −100, 248) extra descendants, while each identically exposed melanized founder cell produced a larger number of extra descendants: 152 (range: 44, 278). This difference was statistically significant (p = 0.038 using the Mann-Whitney U test and 0.030 using the 2-tailed Student’s t-test).

At sufficiently high dose rates (above 5000 mGy/h), the effect of radiation was reversed from growth enhancement to growth inhibition, and proliferation of *C. neoformans* was slowed down (Table 1 and Figures 2–4).

### Insight from Mathematical Modeling

**Figure 5:**
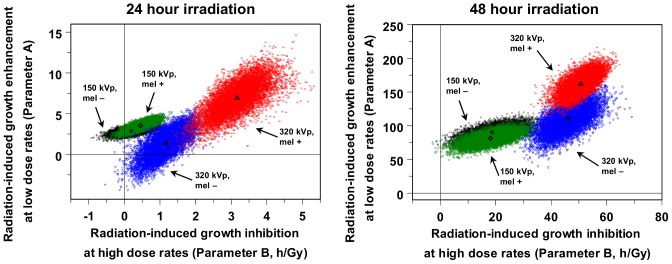
Analysis of best-fit model parameter values. The proposed model, Q = A(e, t, m) – [B(e, t, m) x R], which predicts the radiation effect on proliferation relative to background conditions (Q), based on radiation dose rate (R), X-ray peak energy (e), duration of irradiation (t), and cell melanization status (m), was fitted to 10,000 Monte Carlo simulated data sets, generated from each observed data set by nonparametric bootstrapping. This procedure was repeated for 8 observed data sets, which represent combinations of 150 or 320 kVp X-ray peak energy, 24 or 48 hour exposure duration, and positive or negative cell melanization status (mel+or mel –). Each “cloud” of points represents the spread of values for model parameters A and B which are consistent with the corresponding data set, randomly varied by bootstrapping. Black open symbols superimposed on each “cloud” represent best-fit parameter values to the observed data (unperturbed by bootstrapping).

Our quantitative model, Q = A(e, t, m) – [B(e, t, m) x R], was fitted separately to each of 8 experimentally observed data sets, which represent the 8 combinations of X-ray peak energy (150 or 320 kVp), exposure duration (24 or 48 hours), and cell melanization status (melanized or non-melanized). The best-fit model predictions corresponding to each of these data sets are shown as lines in Figures 3–4. To assess how sensitive these predictions were to random fluctuations in the data, the model was fitted to multiple Monte Carlo simulated data sets generated from each experimentally observed data set by nonparametric bootstrapping (as described in Materials and Methods). The distributions of values for parameters A and B, produced by fitting the model to bootstrapped data, are shown as “clouds” of points in Figure 5. The size and location of the region occupied by each “cloud” indicate the spread of model parameter values which are consistent with the observed data set. The degree of separation between regions which encompass model fits to different data sets indicate how different the best-fit model parameter values corresponding to one data set are from those corresponding to the other data set, and how sensitive these differences are to random variation in the data sets.

Our model had limited ability to describe the data from *C. neoformans* cells irradiated for 24 hours. This conclusion was suggested by: (1) the wide distributions of parameter values produced by fitting the model to bootstrapped data (Figure 5), and (2) the low adjusted R-squared (0.47), and the evidence against normality of residuals (Shapiro-Wilk p-value <10^−4^), produced by fitting the model to experimentally observed data using stepwise multiple linear regression (described in Materials and Methods). The explanation is that variability in the duration of the lag phase (i.e. of the time from the start of irradiation until cell proliferation begins), which is known to occur in *C. neoformans* cultures with low starting cell concentrations [16], caused large variations in CFU/ml counts after 24 hours. This “noise”, which was amplified exponentially during the period of rapid cell division following the lag phase, masked the relatively subtle effects of X-ray peak energy and cell melanization. Consequently, the best-fit value for parameter A, produced by fitting the model by stepwise multiple linear regression, was common for both melanized and non-melanized cells, because differences between their responses could not be discerned with statistical significance. However, this best-fit value for parameter A was 3.2 (95% CI: 2.3, 4.1), significantly different from zero (2-tailed p-value = 4.3×10^−10^), indicating that the overall growth-enhancing effect of low dose rate irradiation on *C. neoformans* became apparent as early as 24 hours after the start of irradiation.

In contrast, the data set from *C. neoformans* cells irradiated for a longer time (48 hours) was less affected by differences in lag phase duration, because the lag phase constituted a smaller fraction of the total irradiation time, compared with 24 hour exposures. Consequently, applying the model to this data set was more informative: the distributions of parameter values produced by fitting the model to bootstrapped data were relatively narrow (Figure 5), and fitting the model by stepwise multiple linear regression produced a relatively high adjusted R-squared (0.70), with no evidence against normality of residuals (Shapiro-Wilk p-value = 0.81). The effects of X-ray peak energy and cell melanization were detected. Growth enhancement at low dose rates was significantly higher for melanized cells exposed to 320 kVp X-rays, than for all other cell and exposure groups. A common value of parameter A (100, 95% CI: 83, 116) was produced (by stepwise multiple linear regression) for melanized and non-melanized cells exposed to 150 kVp X-rays, and for non-melanized cells exposed to 320 kVp X-rays. However, for melanized cells exposed to 320 kVp X-rays, parameter A was significantly higher, by 55 (95% CI: 29, 81), with a 2-tailed p-value of 4.8×10^−5^. Consequently, this analysis suggested that each melanized founder cell exposed to 320 kVp X-rays produced (on average) 55 more descendants over 48 hours of exposure, than an identically irradiated non-melanized founder cell, or than a founder cell exposed to less energetic 150 kVp X-rays.

Dependence on X-ray peak energy was also suggested for dose rate dependent growth inhibition (parameter B), which, after 48 hours of irradiation, was 24.4 (95% CI: 11.6, 37.2) h/Gy for 150 kVp exposures, and 46.7 (95% CI: 41.3, 52.1) h/Gy for 320 kVp exposures. However, these differences in parameter B should be regarded with caution because the range of tested dose rates for 150 kVp X-rays did not include values >5000 mGy/h, i.e. those dose rates at which *C. neoformans* growth inhibition was strongest. We intend to investigate the dose rate and energy dependences of growth inhibition in more detail in future studies.

These applications of the model again suggested that: (1) proliferation of *C. neoformans* is enhanced by low dose rates of both 150 and 320 kVp X-rays, and this is most clearly visible after 48 hours of continuous irradiation, (2) the effect of melanin on the radiation response of *C. neoformans* cells exposed to 150 kVp X-rays is not discernible, (3) higher energy (320 kVp) X-rays, however, are clearly more effective in stimulating the proliferation of melanized *C. neoformans* cells, compared with non-melanized cells.

## Discussion

By combining quantitative mathematical modeling with Monte Carlo data simulation techniques, and applying them to experimental data on the effects of X-ray dose rates spanning six orders of magnitude, at two different X-ray peak energies, on genetically identical fungi which differ only by melanization status, our study provides new insight into the influence of chronic irradiation on fungal proliferation. The finding that low dose rates of ionizing photons (X-rays) stimulate the growth of certain fungi in a dose rate independent manner (i.e. the magnitude of the growth enhancement is constant over a wide range of dose rates) suggests that chronic irradiation induces a qualitative (rather than a quantitative) shift in cell homeostasis, e.g. due to activation (or release from inhibition) of certain metabolic and cell cycle-related pathways. Interestingly, the observed stimulation of fungal growth by X-rays seems to be independent of the carbon source in the growth medium, as in this study the growth enhancement was observed with acetate as a carbon source while previous observations were made when fungi were grown with sucrose [8].

Melanin plays a subtle, but important role in modulating the phenomenon of radiation-induced growth enhancement. This finding is consistent with the higher survival of melanized vs. non-melanized fungi after high-dose acute irradiation [13], and also with data from radioactively-contaminated environments, where melanized fungal forms tend to be over-represented in comparison with non-melanized ones [2], [7], [9], presumably because even a small competitive advantage provided by melanin would be sufficient for melanized forms to dominate over the long term.

Intriguingly, our study suggests that effects of melanin on fungal proliferation are X-ray peak energy dependent, with more energetic X-rays (320 kVp) producing a greater growth-stimulatory effect on melanized *C. neoformans* cells, than less energetic (150 kVp) ones. The mechanisms for this energy dependence are as yet unknown. Prior reports have shown that ionizing radiation alters the oxidation-reduction behavior of melanin [15], and that melanin affects ATP levels in irradiated cells [12]. Perhaps these interactions between melanin and ionizing photons are photon energy dependent, and we intend to investigate this in future studies.

It will also be worthwhile to examine and quantitatively model the effects of chronic irradiation and melanin on genetic and epigenetic regulation in fungi, particularly under stressful conditions of nutrient limitation and/or suboptimal temperature, which are relevant for fungal ecology in radiation-contaminated areas.

## Materials and Methods

### *C. neoformans* Culturing and Irradiation

*Cryptococcus neoformans* (strain H99) was cultured in liquid medium for 4 days. Cells grown in medium containing 1 mM L-DOPA became melanized, while those grown without L-DOPA remained non-melanized. Immunofluorescence of melanized cells was performed using FITC-conjugated melanin-specific antibody 11B11, which only binds to pigmented cells.

Melanized and non-melanized cells, at a starting concentration of approximately 1000 colony-forming units per milliliter (CFU/ml), were irradiated in darkness at 25+/−1° C for 24 and 48 hours in minimal medium [14], with 1 mM acetate as a carbon source, and plated for colony formation. Irradiations were performed twice, with 4 or 6 samples for each condition in each exposure time, using the X-RAD 320 Biological Irradiator (Precision X-ray, Inc.). Al, Cu, and Sn filters and Pb shielding allowed delivery of dose rates ranging from 0.002 milli-Gray/hour (mGy/h) to 5500 mGy/h of X-rays with peak energies of 150 and 320 peak kilovoltage (kVp). The filtering and shielding remove the least energetic photons from the original machine-generated X-ray spectrum, thereby making the energy distribution of photons which reach the cells narrower and closer to the peak energy. The dose rates reaching the cells were calculated using measurements from the ion chamber built into the X-RAD 320 unit (which measures the dose rate after the beam has passed the filter), and from a second ion chamber (clinical model PTW N30013 SN05188) inserted under the lead shielding.

The growth kinetics of *C. neoformans* cultures under experimental conditions began with a lag phase of variable duration (up to 18 hours) during which the cells did not proliferate, followed by exponential growth (most rapid between 24 and 48 hours). Cell concentrations began to saturate after around 72 hours, sometimes reaching 1.0 million CFUs/ml.

### Data Analysis and Mathematical Modeling

Because CFU/ml counts are sensitive to variations in the length of the lag phase, which occur in *C. neoformans* cultures with low starting cell concentrations [16], we quantified the effects of radiation on *C. neoformans* proliferation by calculating the radiation-induced change (Q_e_) in CFU/ml, normalized by the initial cell concentration, as follows: Q_e_ = (X_r_(i) – X_c_)/X_0_, where X_r_(i) is the CFU/ml for each irradiated culture, X_c_ is the mean CFU/ml for corresponding unirradiated controls, and X_0_ is the mean CFU/ml at the start of irradiation. Q_e_ represents the average number of extra descendants produced by each founder cell due to radiation effects on proliferation.

The experimentally-derived Q_e_ values were analyzed by fitting the following mathematical formalism: Q = A(e, t, m) – [B(e, t, m) x R]. Here Q is the predicted radiation-induced change in CFU/ml, normalized by the initial cell concentration, and R is radiation dose rate. The adjustable parameters (A and B) depend on X-ray peak energy (e), duration of irradiation (t), and cell melanization status (m). Parameter A represents radiation-induced growth enhancement (assumed to be independent of dose rate), and parameter B represents radiation-induced growth inhibition (assumed to be proportional to dose rate).

The experimental data were divided into 8 sets, which represent the 8 possible combinations of X-ray peak energy (e), duration of irradiation (t), and cell melanization status (m): (1) non-melanized cells, 150 kVp X-rays, 24 hour exposure duration, (2) melanized cells, 150 kVp X-rays, 24 hour exposure duration, (3) non-melanized cells, 320 kVp X-rays, 24 hour exposure duration, (4) melanized cells, 320 kVp X-rays, 24 hour exposure duration, (5) non-melanized cells, 150 kVp X-rays, 48 hour exposure duration, (6) melanized cells, 150 kVp X-rays, 48 hour exposure duration, (7) non-melanized cells, 320 kVp X-rays, 48 hour exposure duration, (8) melanized cells, 320 kVp X-rays, 48 hour exposure duration. The model, Q = A(e, t, m) – [B(e, t, m) x R], was fitted to each of these 8 data sets separately, generating 8 combinations of best-fit values for parameters A and B.

The effects of possible non-linear dependences of Q on radiation dose rate were tested by adding to the model terms such as R^0.1^, R^0.5^, or R^2^. These terms did not increase the adjusted R-squared values for the model fits to the majority of the 8 data sets. Consequently, they were not included in the model.

The sensitivity of model predictions to perturbations of the observed data were explored by nonparametric bootstrapping [17], using a customized code written in FORTRAN 77. Each of the 8 experimentally observed data sets contained a certain number of “elements”, where each element represented a combination of X-ray dose rate (R) and its effect on cell proliferation (Q_e_). A perturbed data set was created by randomly selecting (with equal probability) elements from the observed data set. Thus, in the perturbed data set some elements could be repeated, while others could be absent, but the total number of elements remained the same as in the original observed data set. The model, Q = A(e, t, m) – [B(e, t, m) x R], was fitted to the perturbed data set, producing a combination of parameters A and B. This procedure was repeated 10,000 times on each of the 8 experimental data sets, generating distributions of parameters A and B.

Stepwise multiple linear regression [18] was used to assess the ability of the model to describe the data, and to investigate the effects of individual predictors such as X-ray peak energy and cell melanization status. Briefly, the procedure consisted of adding to the model a new predictor only if it significantly improved the model fit (i.e. forward selection, using a p-value threshold of <0.05), and removing predictors from the model if doing so did not reduce the fit quality significantly (i.e. backward elimination, using a p-value threshold <0.1). These steps were continued until only those predictors which significantly improved the model fit remained in the model. The regression was performed independently on data from 24 and 48 hour irradiations.
